# Terahertz beam switching by electrical control of graphene-enabled tunable metasurface

**DOI:** 10.1038/s41598-017-14493-8

**Published:** 2017-10-26

**Authors:** Yin Zhang, Yijun Feng, Junming Zhao, Tian Jiang, Bo Zhu

**Affiliations:** 1 Department of Electronic Engineering, School of Electronic Science and Engineering, Nanjing University, Nanjing, 210093 China; 2 Jiangsu Provincial Key Laboratory of E-Business, Nanjing University of Finance and Economics, Nanjing, 210003 China; 3 Institue of Food Economics, Nanjing University of Finance and Economics, Nanjing, 210003 China

## Abstract

Controlling the terahertz wave, especially the dynamical and full control of terahertz wavefront, is highly demanded due to the increasing development of practical devices and application systems. Recently considerable efforts have been made to fill the ‘terahertz gap’ with the help of artificial metamaterial or metasurface incorporated with graphene material. Here, we propose a scheme to design tunable metasurface consisting of metallic patch array on a grounded polymer substrate embedded with graphene layers to electrically control the electromagnetic beam reflection at terahertz frequency. By adjusting geometric dimension of the patch elements, 360 degree reflection phase range may be achieved, thus abrupt phase shifts can be introduced along the metasurface for tailoring the reflected wavefront. Moreover, the reflective phase gradient over the metasurface can be switched between 90 and 360 degree by controlling the Fermi energy of the embedded graphene through voltage biasing, hence dynamically switching the reflective beam directions. Numerical simulations demonstrate that either single beam or dual beam dynamically switching between normal and oblique reflection angles can be well attained at working frequency. The proposed approach will bring much freedom in the design of beam manipulation devices and may be applied to terahertz radiation control.

## Introduction

Manipulating electromagnetic (EM) waves in a tunable manner is highly desired in both fundamental research and practical application. Recently, the two-dimensional version of metamaterial, termed as metasurface that consists of an ultrathin metallic/dielectric structure, has attracted considerable attentions due to its extraordinary ability of controlling the EM waves^1–5^. The wavefront transmitted or reflected from a metasurface does not only rely on gradual phase changes accumulated along the optical path, but also depends on abrupt phase shifts over the scale of the wavelength attained by spatially tailoring the geometry and planar arrangements of the meta-particles composing the metasurface^1^. Based on this peculiar property, metasurface can mold the wavefront of the reflected and refracted beams in nearly arbitrary ways and provide complete manipulation of EM waves^6–10^. Although metasurface concept has provided a new degree of freedom to mold the EM wave flow, most metasurfaces designed for various applications lack of tunable response as they are composed of passive building blocks (or called the meta-particles) and their EM responses are relying solely on the geometry and arrangements of the meta-particles. However, there are increasing requirements for the reconfigurability of manipulating electromagnetic radiation, as well as the dynamical control of the responses of EM components for the practical applications^11^. Graphene, the newly discovered two-dimensional material, has been proved to be a good candidate to meet the requirement of EM property reconfigurability, especially at the terahertz (THz) frequencies. The emerging field of graphene now finds a variety of potential applications, such as subwavelength waveguides, high-sensitive detectors, as well as tunable metamaterials^12–16^. Considerable attentions have been focused on using graphene to design tunable THz metamaterials^17–29^. Among the different tuning schemes for THz devices, graphene has revealed its potential application in high-performance tunable terahertz devices, since it produces a largely-tunable surface conductivity with respect to the external electrostatic biasing^30–37^.

Apart from those preliminary investigations on graphene based THz tunable metamaterials, more fascinating physical properties and potential applications need to be further explored. For example, the phase modulation in the terahertz region still remains as a major challenge^38^. Most of early THz phase modulators were based on liquid crystals^39^ or semiconductor quantum well^40^, which resulted in limited working conditions of low-temperature and low-speed. Recently, more and more efforts have been devoted to develop room temperature solid-state THz phase modulator with tunable metamaterials, where electrical controls have been realized by a carrier injection/depletion scheme in a Schottky diode^41^ or by gated graphene^17^. Although some THz phase modulators have been demonstrated, the resulted phase shifts are still insufficient to completely control the wavefront^42^. When utilizing metasurface concept to mold the wavefront, full phase control by the resonant meta-particle is required. Therefore, developing tunable metasurface with full range of phase response becomes an important issue for dynamical and full control of THz wavefront, as well as radiation beam directions in many applications.

Here, in this paper we present a scheme to achieve dynamical switch of THz reflection beam through tunable metasurface which employs a traditional metal/dielectric metasurface embedded with gated graphene layers. We firstly design a traditional reflective metasurface that can produce abnormal reflection of terahertz beam, which consists of meta-particles of differently sized metallic patches with rectangular slot on top of a grounded polymer substrate to achieve certain phase gradient. Then a gated structure that is composed of double graphene layers sandwiched with a thin silicon dioxide insulating film is embedded into the polymer substrate to function as a reflection/transmission amplitude modulator. The graphene enabled tunable metasurface could dynamically switch the reflection direction of either single beam or double beams. By adjusting the bias voltage on the double graphene layers, the total phase shifts over the metasurface can be switched between 2π and π/4, and thus the reflection beam direction can be controlled. As proof-of-concept examples, we have designed different samples to achieve either single reflection beam or double reflection beam switching between different directions. Numerical simulations reveal that multi-angle beam steering is obtained at around 1 THz within a theoretical switching time in the pico-second range. The proposed metasurface may offer high-speed THz modulation scheme for a variety of potential applications, such as spatial phase modulation, beam scanning or shaping, as well as imaging system. Moreover, as we employ non-structured graphene layers as controlling element, the proposed metasurfaces may provide feasible examples of bridging the theory and possible experimental realizations in tunable graphene devices.

## Results

### Metasurface for anomalous THz reflection

The phase shift of a resonant element between incident wave and radiation wave depends on the resonant characteristics. Through tailoring meta-particle geometry of the metasurface, the resonance frequency will accordingly change, resulting in shifting of the phase dispersion curve. To achieve an abnormal reflecting beam with a reflective metasurface, a phase gradient covering the full range of 2π should be created by arranging different sized meta-particles through a uniformly varied sequence in a two-dimensional plane. The abnormal reflection angle is determined by the generalized Snell’s law expressed as^1^:

1 $$\sin ({\theta }_{r})-\,\sin ({\theta }_{i})=\frac{{\lambda }_{0}}{2\pi {n}_{i}}\frac{d{\rm{\Phi }}}{dx},$$

where *θ*_*i*_ or *θ*_*r*_ is the angle of incidence or reflection respectively, *λ*_0_ is the wavelength in free space, *n*_*i*_ is the refractive index of the incident medium, and *d*Φ/*dx* represents the phase gradient on *dx* range. If the electromagnetic wave is perpendicularly incident on the metasurface from the air, (1) can be simplified as:

2 $${\theta }_{r}=\arcsin (\frac{c}{2\pi f}\frac{d{\rm{\Phi }}}{dx}),$$

where *c* and *f* are the speed of light in free space and frequency of the incident wave, respectively. At a certain frequency, the abnormal reflection angle *θ*_*r*_ merely depends on *d*Φ/*dx*.

**Figure 1:**
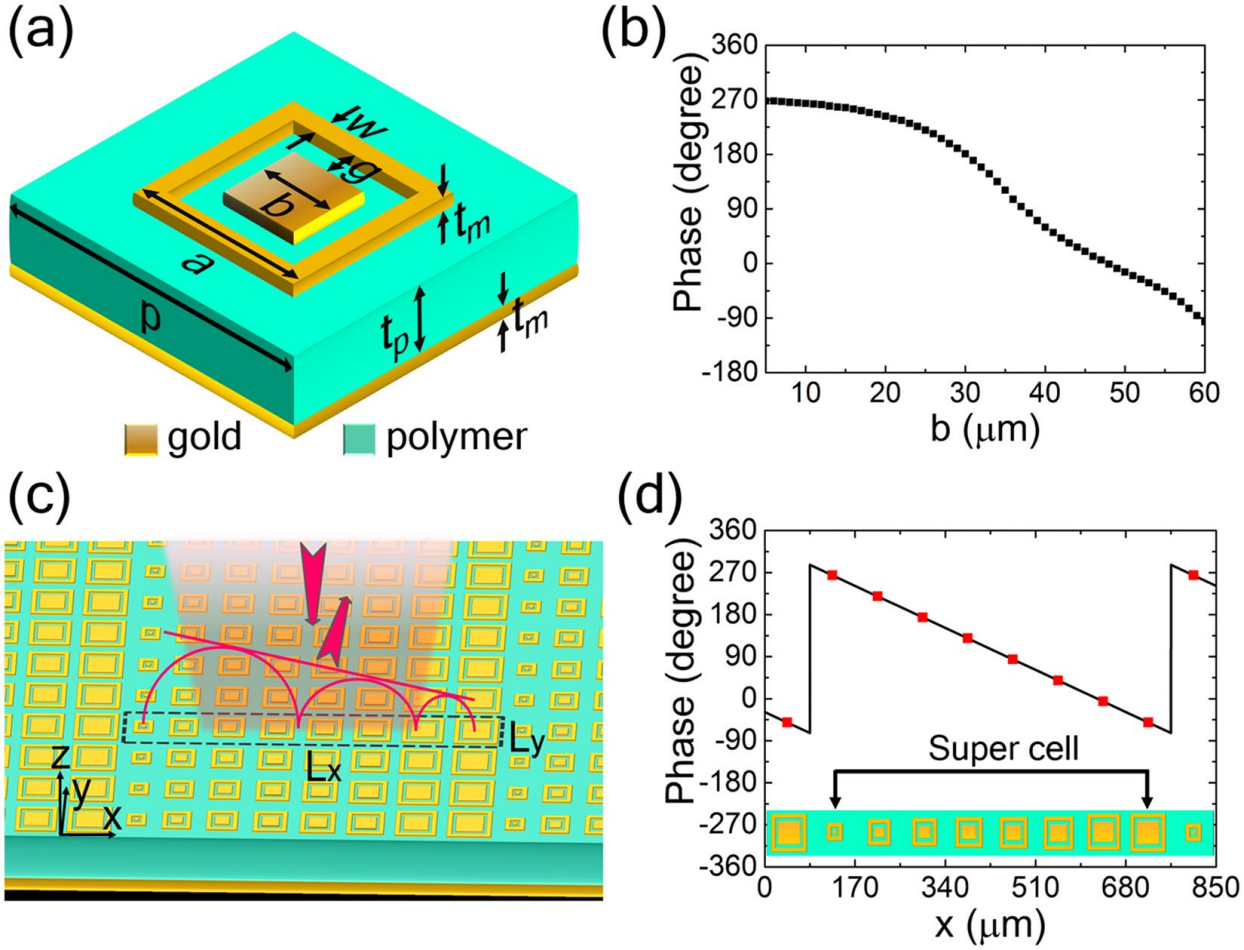
Geometry and working mechanism of the anomalous reflection metasurface. (**a**) Schematics of the designed unit cell. (**b**) The reflection phase at 1 THz as a function of *b* for terahertz wave incidence. (**c**) A super cell in the sample (region surrounded by dashed line) consists of 8 unit structures with top Au square patch-loop-structure. (**d**) Reflection phase of each unit structure within a super cell, with solid line representing relationship between phase and position. The dimension *b* of eight particular meta-particles are 10 μm, 25 μm, 31 μm, 35 μm, 38 μm, 43 μm, 49 μm, 56 μm, respectively.

The proposed metasurface structure is schematically depicted in Fig. 1, with a unit cell (meta-particle) shown in Fig. 1(a) and a supercell in the dashed box in Fig. 1(c). Here a gold ground plane is used ensuring a reflective metasurface, which couples with the gold square patch-loop-structures on the upper layer via a TOPAS polymer spacer (which is chosen due to its low absorption and stable refraction index (about 1.53) across the THz band)^43^. The geometrical parameters of the unit cell are set after performance optimization through full wave EM simulation. The period *p* of the unit cell and the thickness *t*_*p*_ of the polymer layer are 85 μm and 30 μm, respectively. The metallic layers including the square patch-loop-structures and the ground plane have a thickness *t*_*m*_ of 0.2 μm. Both the line width *w* and the gap *g* of square patch-loop-structure are fixed as 5 μm. The side length *b* of the internal patch is a variable parameter and changed from 5 μm to 60 μm to obtain the desired linear reflection phase responses. Evidently the length *a* is related to *b* by *a* = *b* + 2*w* + 2 *g*. As a result, it is able to obtain a roughly linear variation of the phase shift in a large range (0 to 2π) by tuning the patch length *b* at the working wavelength *λ* = 300 μm (see Fig. 1(b)). Furthermore, the performance of designed metasurface should be polarization independent due to the C4 symmetric of the unit cell structure. As an example, eight particular structures are selected for the meta-particles to construct the super cell of the metasurface with the length *b* gradually increasing along *x* direction while the reflection phase decreasing as illustrated in Fig. 1(d). Such super cell in the metasurface could ensure an anomalous reflection in *xoz*-plane. For this particular design, the generalized Snell’s law predicts that a normally incident terahertz wave will be redirected to a scattering beam along an angle *θ*_r_ = 26° (*d*Φ* = *π/4, *dx = p*) after reflection by this metasurface.

**Figure 2:**
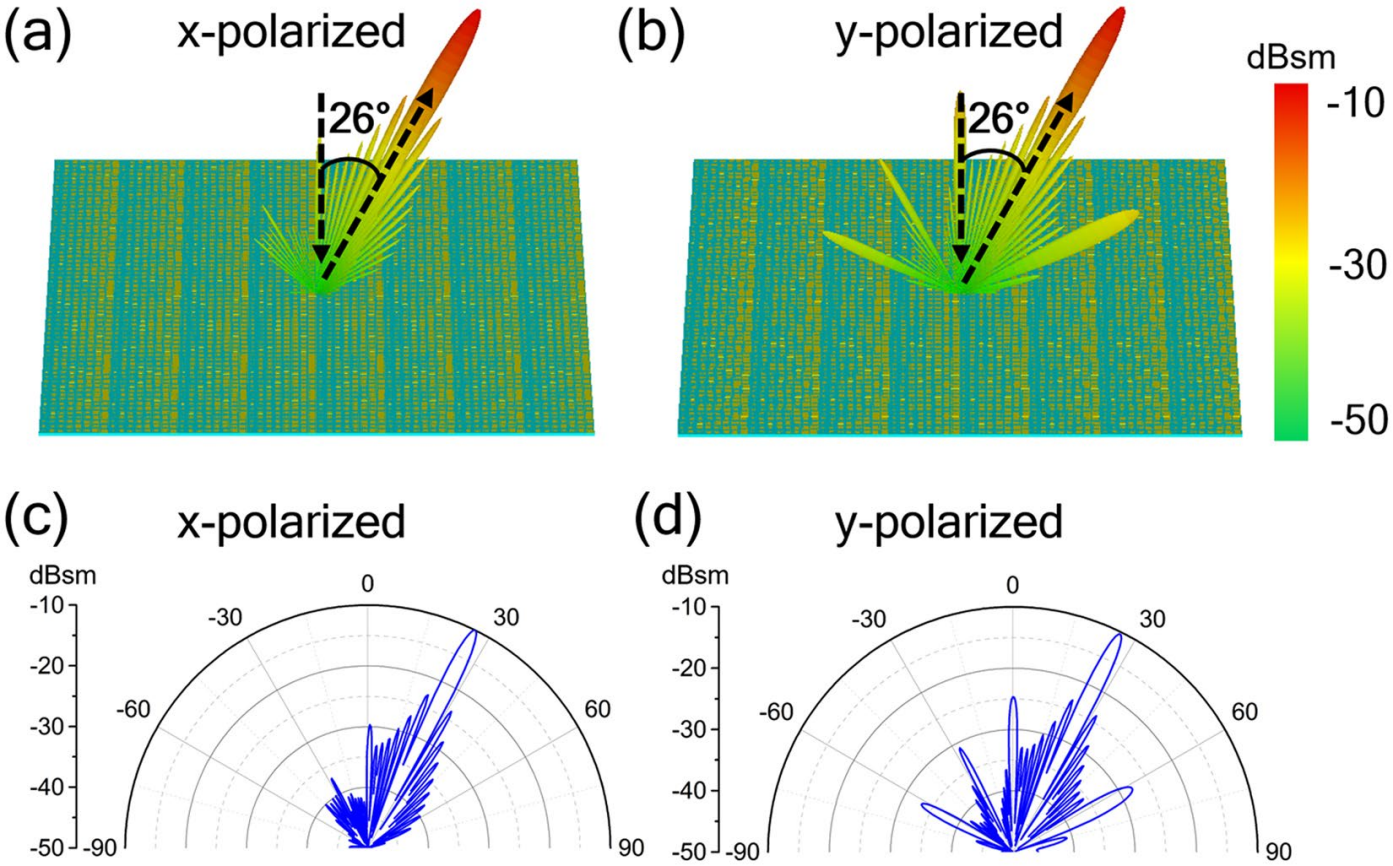
Simulated far-field scattering patterns. (**a**,**b**) 3D far-field scattering patterns, and (**c**,**d**) 2D far-field scattering patterns in polar coordinates for the anomalous reflection metasurface under normal incidence of (**a**,**c**) *x*-, and (**b**,**d**) *y*-polarization at 1.0 THz.

Assuming a plane wave normally impinges on the metasurface, the scattering performance of the metasurface is verified by full-wave numerical simulations. Figure 2 shows the three-dimensional (3D) far-field radar cross section (RCS) distribution and two-dimensional (2D) scattering patterns in the *xoz*-plane of the metasurface with normal incidence at 1 THz, respectively. As illustrated in Fig. 2(a–d), the normal incident wave is anomalously reflected along the angle of 26°, which is in good agreement with the theoretical prediction based on the generalized Snell’s law. This anomalous reflection peak caused by the phase gradient along *x* direction on the metasurface indicates a significant suppression of the normal (or specular) reflection. In addition, the similar reflection performance for either *x*- or *y*-polarized incidence demonstrates that the anomalous THz wave reflection by the proposed metasurface can be achieved for both polarized waves.

### Single reflection beam switching

**Figure 3:**
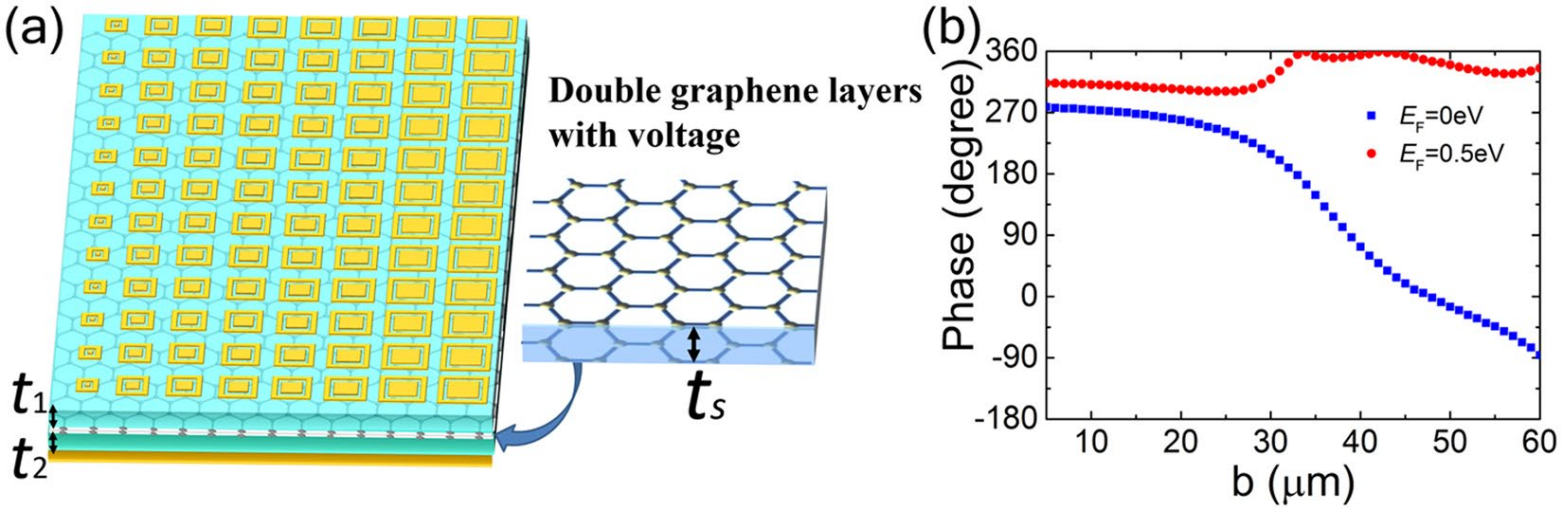
Single-beam switching metasurface. (**a**) Schematics of the metasurface, with right part representing the gated structure consisting of double graphene layers sandwiched with a thin silicon dioxide insulating film. (**b**) The reflection phase of the unit cells as functions of the dimension *b* at 1.0 THz for *E*_F_ = 0 eV (blue square) and *E*_F_ = 0.5 eV (red filed circle), respectively.

Based on the previous metasurface design for anomalous reflection, we propose a tuning scheme for single THz reflection beam switching with graphene used as controlling element, as schematically shown in Fig. 3(a). The voltage-tunable gated structure, composed of double graphene layers separated with a thin silicon dioxide layer, is embedded into the polymer substrate to dynamically modulate the THz wave. The dimension parameters (*p*, *t*_*p*_, *t*_*m*_, *w*, *g*) of the top gold structures are consistent with that in Fig. 1. The silicon dioxide layer with a thickness of *t*_s_ of 0.1 μm is set apart with a distance *t*_1_ of 8 μm from the top metallic film, and a distance *t*_2_ of 21.9 μm from the ground plane film.

As indicated by the surface conductivity of graphene, terahertz transmission in graphene can be modulated by tuning its electrical conductivity or Fermi level (*E*_F_)^31–33^. The modulation device usually consists of single or multiple capacitively coupled graphene-graphene pairs, so that the transmission/reflection of THz wave can be controlled by electrically tuning the Fermi level in graphene via DC biasing voltage^31^. At zero bias (corresponding to *E*_F_ = 0 eV), the Fermi level is at the Dirac point of all graphene layers resulting in near-unity THz wave transmission. With the bias voltage increasing (corresponding Fermi energy *E*_F_ increasing), the reflection from the graphene layers will increase but transmission will decrease. When the bias voltage increases to a certain level (for example, corresponding to *E*_F_ = 0.5 eV), the reflection will be significantly higher than the transmission. Therefore, the reflection phase of the unit cell can be changed noticeably by biasing on graphene layers. Figure 3(b) depicts the reflection phase change for the unit cells with different size *b* at *E*_F_ = 0 eV and *E*_F_ = 0.5 eV, respectively. It indicates that the phase shift range over the super cell can still achieve full 2π at zero bias but will reduce to π/4 with an applied bias. This insures that when the graphene-graphene pair is unbiased the metasurface can keep original phase gradient and achieve similar abnormal reflection, while as the graphene-graphene pair is biased, the phase gradient is almost vanished and the metasurface will produce specular reflection. As a consequence, by applying voltage bias to the gated structure, the phase gradient on the metasurface can be changed, and the direction of the radiated beam can be switched. There will be some absorption due to the loss in double graphene layers, but the metasurface can still generate considerable reflection to achieve the beam switching.

**Figure 4:**
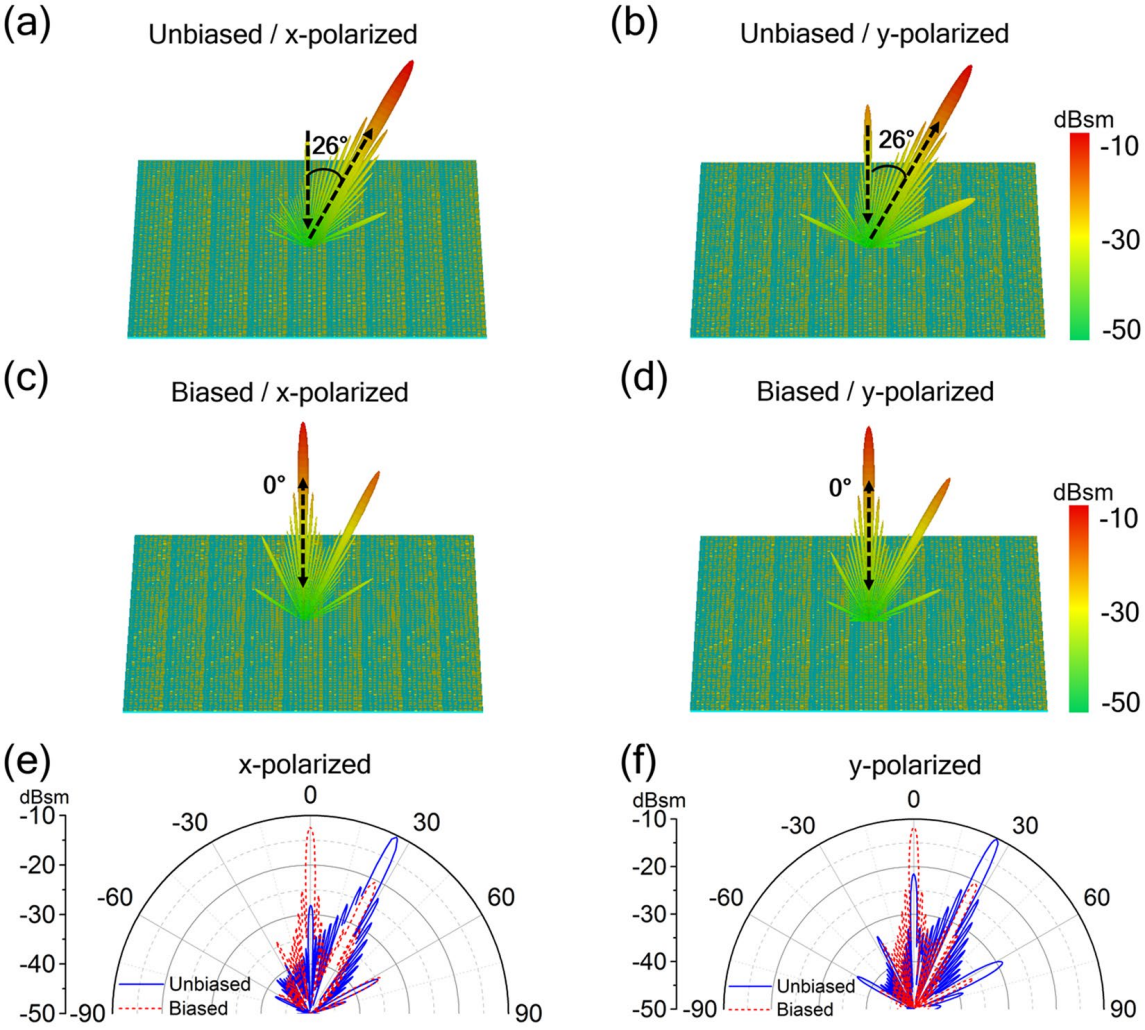
Simulated far-field scattering patterns. 3D far-field scattering patterns in the (**a**,**b**) unbiased and (**b**,**d**) biased state, (**e**,**f**) 2D Far-field patterns given in polar coordinates for two voltage states of the single-beam switching metasurface under normal incidence of (**a**,**c**,**e**) *x*- and (**b**,**d**,**f**) *y*-polarization at 1.0 THz.

Considering the perturbation of the embedded graphene layers, we slightly adjust the geometry of the eight particular meta-particles to maintain uniform phase gradient in the super cell. Therefore, the dimension *b* of eight meta-particles are now taken as 10 μm, 28 μm, 33 μm, 36 μm, 39 μm, 42 μm, 47 μm, 54 μm, respectively. The whole metasurface is formed with 9 super cells periodically arranged along *x* direction, thus it contains 72 × 72 meta-particles (unit cells). Figure 4 shows the 3D and 2D scattering patterns of the metasurface under normal incidence of *x*- and *y*-polarized wave at 1 THz. We clearly observe distinct single main beam switching upon voltage biasing. When the metasurface works in un-biased state, the phase gradient along the metasurface leads to a reflection main lobe at about 26°, which perfectly matches with the theoretical prediction based on the generalized Snell’s law (*d*Φ* = *π/4, *dx = p*, *θ*_r_ = 26°). When applying a gate voltage on the graphene double layers, the phase shifts along the metasurface become flat and the incident wave is mostly reflected to the normal (backward) direction. In the 2D scattering patterns the changes of RCS at both reflected directions are beyond 10 dBsm, indicating a significant single beam switching performance in *xoz*-plane. Moreover, the switchable function can be achieved for both polarized waves as verified by these RCS results.

**Figure 5:**
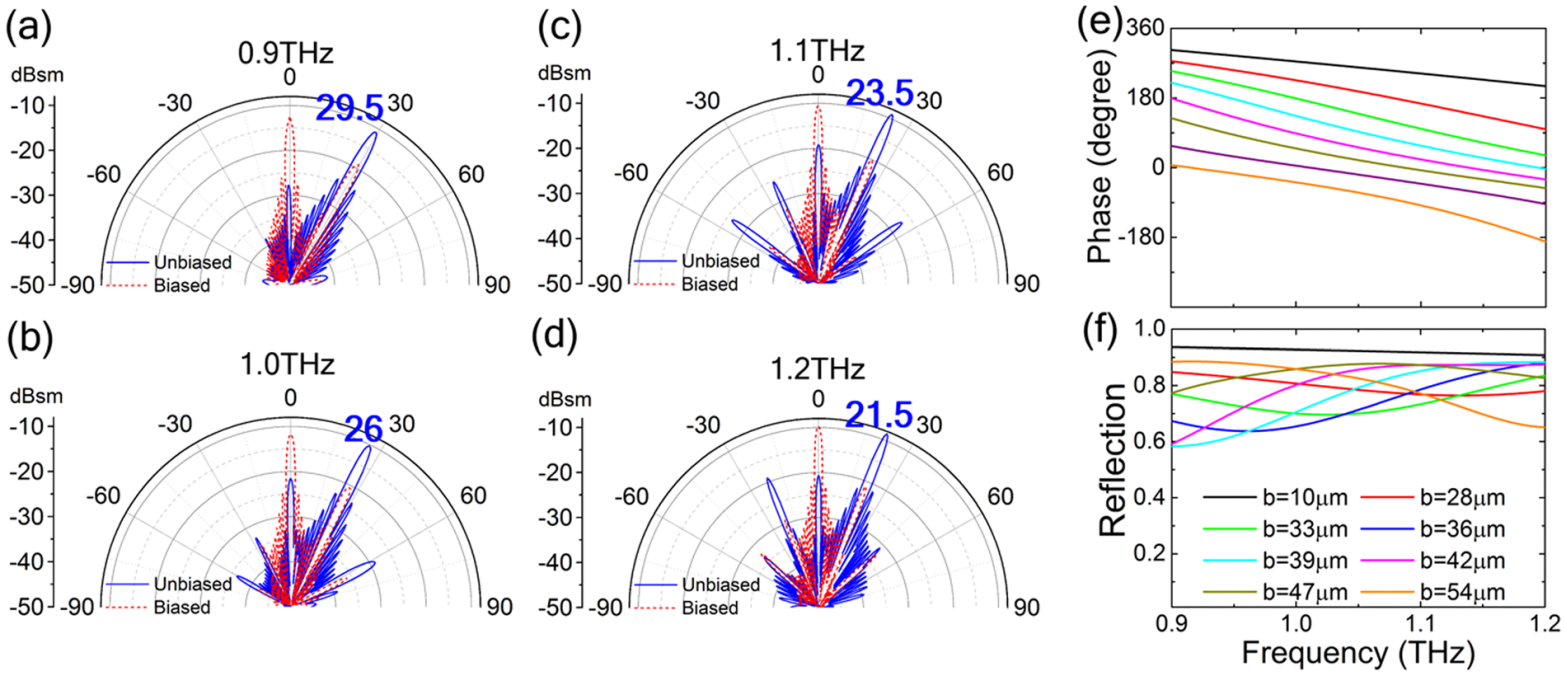
Frequency dependences of the far-field scattering patterns. (**a**–**d**) 2D Far-field patterns given in polar coordinates for two voltage states of the single-beam switching metasurface under normal incidence of *y*-polarized wave at 0.9, 1.0, 1.1, 1.2 THz, respectively. Frequency dependent (**e**) reflection phase and (**f**) amplitude of the eight meta-particles under the normal *y*-polarized plane wave illumination when *E*_F_ = 0 eV. (The blue number indicates the direction angle of the switched beam).

In order to demonstrate the broadband performance of the metasurface, we only consider the illumination of normal incident *y*-polarized plane wave as an example due to the polarization independence of the metasurface. Figure 5(a–d) exhibits the 2D scattering patterns of the metasurface at 0.9, 1.0, 1.1, 1.2 THz, respectively. We can clearly see distinct single main beam switching upon voltage biasing at all working frequencies. When applying a gate voltage on the metasurface, the performance of specular reflection is basically frequency-independent. However, when the metasurface works in unbiased state, the incident wave is mostly reflected along an abnormal angle which slightly decreases with the increase of the frequency. This is due to that the entire phase gradient remain about the same in the working band from 0.9 to 1.2 THz as shown in Fig. 5(e), but the reflection angle *θ*_r_ is negatively correlated with the frequency according to (2) based on the generalized Snell’s law. In addition, as frequency increases, the anomalous reflection peak mildly increases due to the entire reflection amplitude of each meta-particle increases with the increase of the frequency as shown in Fig. 5(f). However, the proposed metasurface can achieve significant beam switching function over a broad frequency bandwidth, thus the switchable abnormal angle would be continuously tuned by adjusting the working frequency.

**Figure 6:**
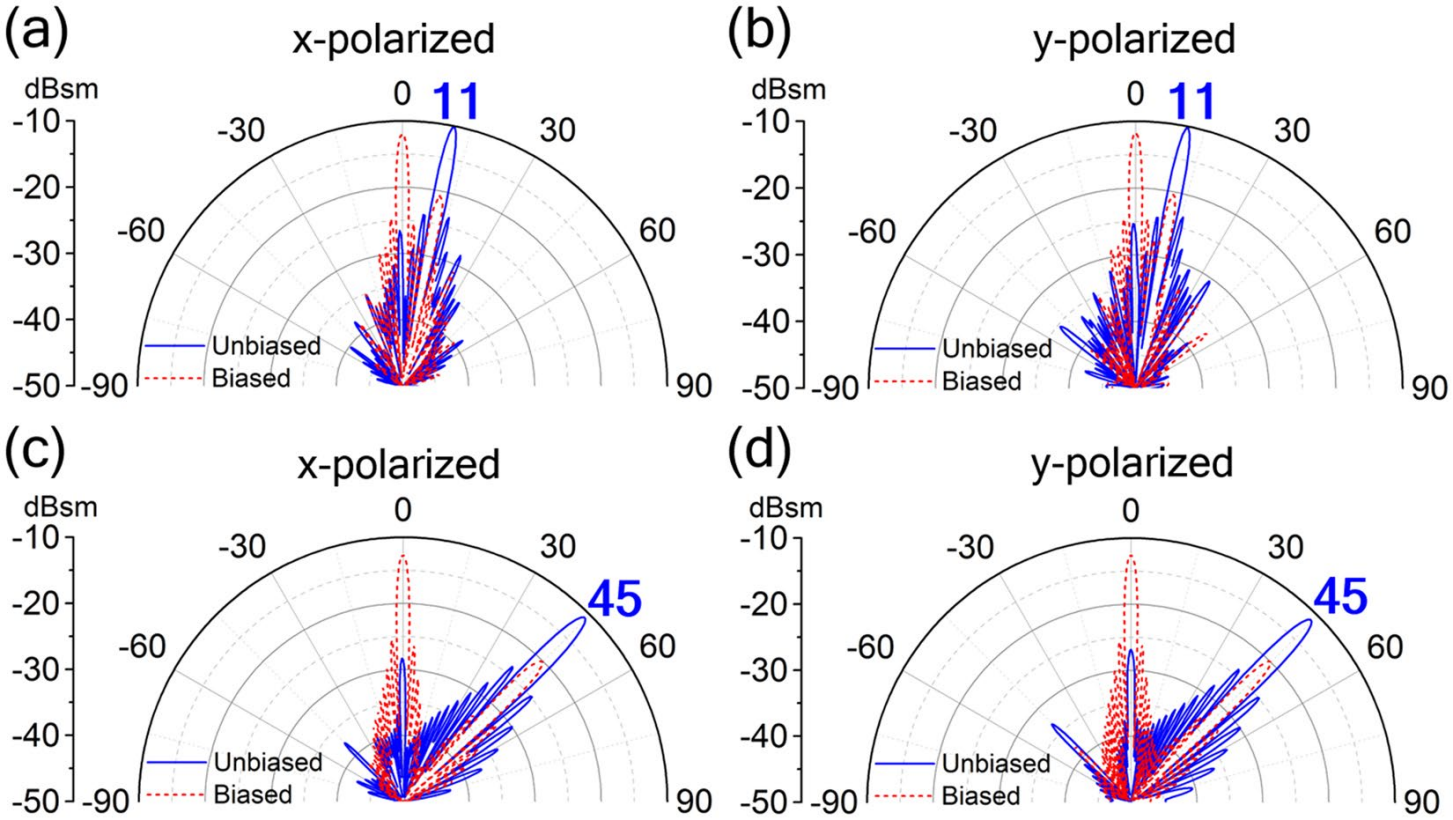
Metasurface designs for different single beam switching. (**a**,**b**) 2D Far-field patterns of the single-beam switching metasurfaces given in polar coordinates indicating beam switch between 0° and 11°, (**c**,**d**) or beam switch between 0° and 45° under normal incidence of (**a**,**c**) *x*- and (**b**,**d**) *y*-polarization at 1.0 THz. (The blue number indicates the direction angle of the switched beam).

The proposed scheme also has good flexibility for achieving different reflection beam directions. Figure 6 exhibits two more metasurface designs with different super-cells, one consists of eighteen particular meta-particles (*b* = 10 μm, 22 μm, 26 μm, 29 μm, 32 μm, 33 μm, 35 μm, 36 μm, 37 μm, 38 μm, 40 μm, 41 μm, 43 μm, 46 μm, 49 μm, 52 μm, 55 μm, 58 μm, other parameters are the same as that in Fig. 3) while another has five particular meta-particles (*b* = 10 μm, 31 μm, 36 μm, 41 μm, 50 μm, other parameters are the same as that in Fig. 3) with gradually increasing length *b* along *x* direction. As shown from the 2D far-field scattering patterns in Fig. 6, for zero voltage states, the two metasurfaces present two different anomalously reflected angles. We note that the first example (Fig. 6(a) and (b)) can achieve excellently single beam switching between 0° (*d*Φ*/dx* ≈ 0, *θ*_r_ = 0°) and 11° (*d*Φ = π/9, *dx* = *p*, *θ*_r_ = 11°), while the second one (Fig. 6(c) and (d)) can switch the reflection beam between 0° (*d*Φ*/dx* ≈ 0, *θ*_r_ = 0°) and 45° (*d*Φ = 2π/5, *dx* = *p*, *θ*_r_ = 45°), both under normal incidence of *x*- and *y*-polarized wave of 1.0 THz. Actually, the switchable anomalous reflection angle can be correspondingly increased/decreased by increasing/decreasing the phase-gradient (*d*Φ/*dx*) in the super-cell with 2π phase shifting.

### Dual reflection beam switching

**Figure 7:**
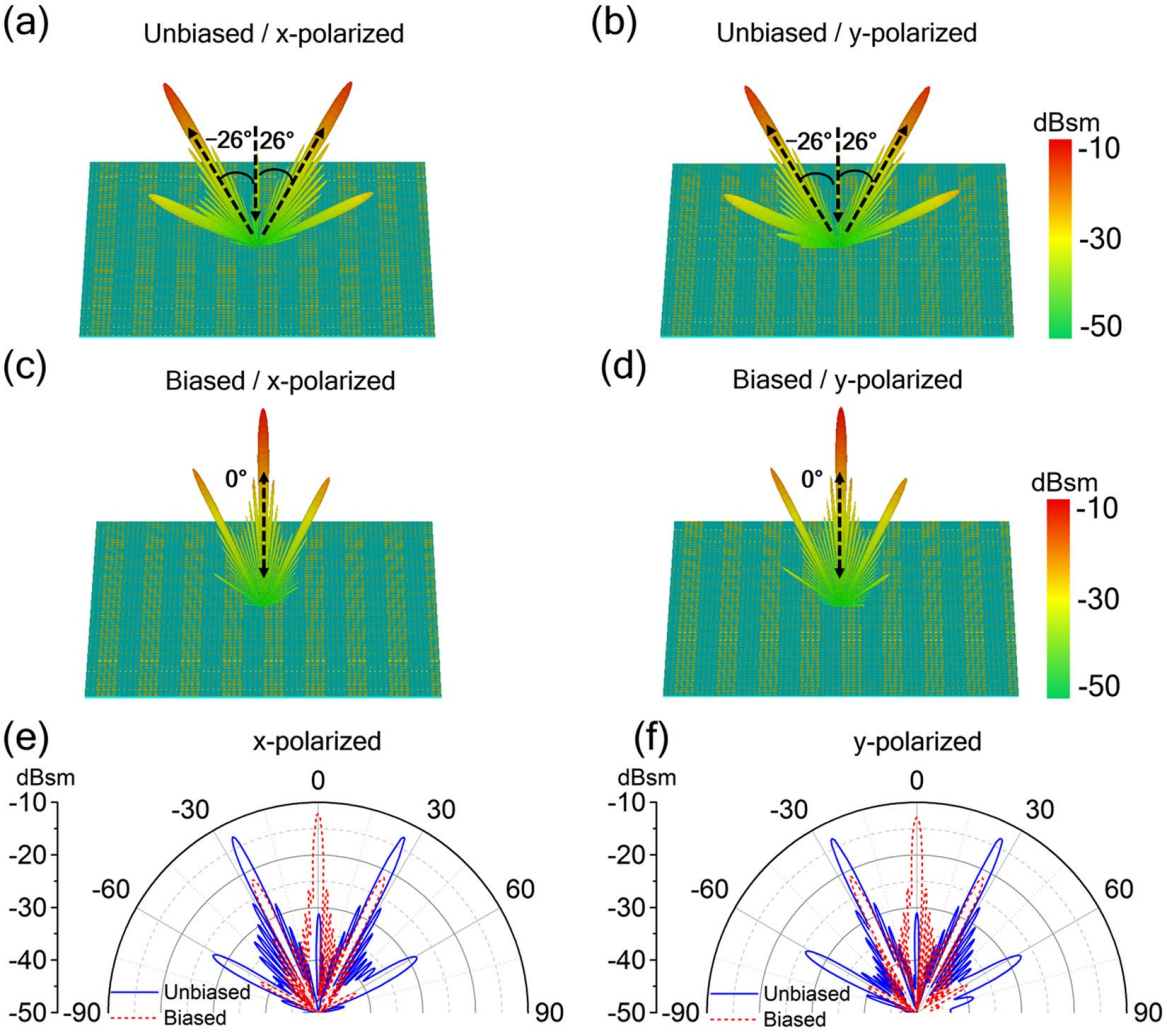
Metasurface designs for dual reflection beam switching. 3D Far-field scattering patterns in the (**a**,**b**) unbiased (main lobe at direction angles of ± 26°) and (**c**,**d**) biased (main lobe at 0° direction angles) state, as well as (**e**,**f**) 2D Far-field patterns given in polar coordinates for two voltage states of the dual beam switching metasurface under normal incidence of (**a**,**c**,**e**) *x*- and (**b**,**d**,**f**) *y*-polarization at 1.0 THz.

**Figure 8:**
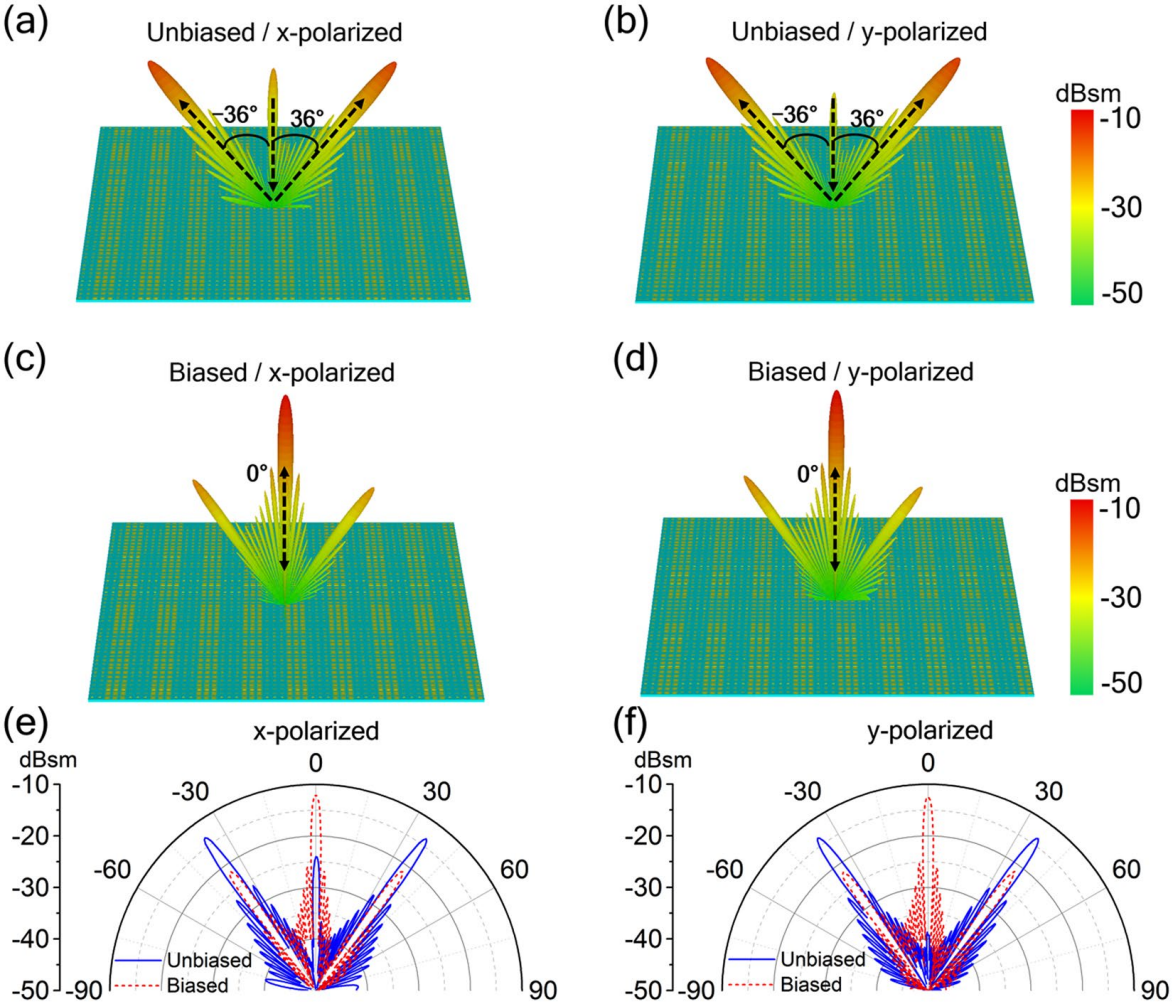
Metasurface designs for dual reflection beam switching. 3D Far-field scattering patterns in the (**a**,**b**) unbiased (main lobe at direction angles of ±36°) and (**c**,**d**) biased (main lobe at 0° direction angles) state, as well as (**e**,**f**) 2D Far-field patterns given in polar coordinates for two voltage states of the dual beam switching metasurface under normal incidence of (**a**,**c**,**e**) *x*- and (**b**,**d**,**f**) *y*-polarization at 1.0 THz.

The proposed design method can be expand to metasurface for dual reflection beam switching. In a similar way, we embed the voltage-tunable gated graphene structure into the polymer substrate of the metasurface with double anomalous reflection beams to construct the desired tunable metasurface. As design examples, we use two particular meta-particles whose reflection phases has 180° difference at working frequency (assuming at 1 THz) to form the dual beam switching metasurface through arranging the meta-particles with different sequences. The two meta-particles denoted as “0” and “1” have different dimension *b* of 10 μm and 39 μm respectively, while other geometric parameters are the same as that of the meta-particle described in Fig. 1. The first design example shown in Fig. 7 is realized with the “0” and “1” meta-particles through a sequence of “00001111…” and contains 68 × 68 meta-particles (unit cells), while the second example shown in Fig. 8 is composed of the two meta-particles through “000111…” sequence and contains 70 × 70 meta-particles (unit cells). The 3D and 2D far-field patterns scattered from the first metasurface under normal incidence are calculated and displayed in Fig. 7(a–d) and Fig. 7(e,f), respectively. At zero voltage bias on the double graphene layers, the reflected wave will be cancelled in the backward direction, so the far-field scattering pattern will produce two beams along symmetrically oriented directions at ± 26° in *xoz*-plane, verifying the generalized Snell’s law (*d*Φ* =* ±π, *dx = *4*p*, *θ*_r_ = ±26°). However, with an applied voltage bias, the reflected wavefront is close to a plane wave, so the far-field scattering pattern will produce a main lobe in the normal direction (*d*Φ*/dx* ≈ 0, *θ*_r_ = 0°). The simulation results presented in Fig. 8 show that the second metasurface design can also achieve the similar dual-beam switching function as the first one except the anomalous reflection peak appearing at ± 36° determined by the generalized Snell’s law (*d*Φ* = *±π, *dx = *3*p*, *θ*_r_ = ±36°). Hence, at the working frequency, both proposed metasurfaces prove the functionality of double THz reflection beam switching enabled by graphene, and the beam direction can also be predictably tuned by adjusting arranged sequence of the two particular meta-particles.

## Discussion

To summarize, we have proposed metasurface design to support anomalous reflections for terahertz wave following the generalized Snell’s law. On this basis, we have presented a tuning scheme of the metasurface enabled with graphene gated layers for controlling reflected THz beams. The electrically controllable beam switch capability of the proposed metasurfaces has been successfully demonstrated by numerical simulations on practical realizable structures. The results proved that either single-beam switch operating between normal and other particular reflection angles or the dual-beam switch operating between normal and other particular directions have been achieved at working frequency by controlling the bias voltage on double graphene layers with excellent performance. Moreover, the beam direction can be expediently and predictably controlled by changing the sequence in the super-cell of the metasurface. Therefore, if the reflection phase on the metasurface is gradually distributed along both orthogonal directions through an appropriate two-dimensional sequence, the reflection beam will be switched between normal direction and any desired abnormal orientation in backward half space^3^. The proposed scheme may provide practical opportunity toward the potential applications of graphene for advanced THz wave control devices.

## Methods

### Simulation

Full wave simulations have been carried out to compute the reflection phase and amplitude of meta-particle as well as the far field RCS of the designed metasurface by commercial software, the CST Microwave Studio. In the meta-particle simulation, the unit cell is applied with periodic boundary condition along both *x*- and *y*-directions while open for *z*-direction in the free space. The electric field is applied either parallel to *x*- or *y*-direction to correspondingly obtain the meta-particle performances under the illumination of linearly *x*- or *y*-polarized waves, respectively. In the far-field RCS simulation, the metasurface with finite size is applied with open boundary condition along both *x*- and *y*-directions as well as *z*-direction in the free space. The plane wave excitation with either *x*- or *y*-polarization is utilized to correspondingly obtain the far-field RCS of metasurface under the illumination of linearly *x*- or *y*-polarized plane wave propagating along *z*-direction, respectively. The time domain solver is employed to carry out the specific calculation, then we can analyze the results recorded by the preset field monitors which are set as Far-field/RCS at expected frequencies such as 0.9, 1.0, 1.1, 1.2 THz.

### The conductivity model of graphene and electrically controlling

The graphene monolayer can be electrically modeled as an infinitesimally thin conductive layer characterized by a complex-valued surface conductivity *σ*_s_ (*ω*, *μ*_c_, *Γ*, *T*), where *ω* is the working radian frequency, *μ*_c_ is the chemical potential (i.e. Fermi energy *E*_F_) related to the electrostatic biasing, and *Γ* (*Γ* = *ћ*/2*τ*, *τ* is the electron-phonon relaxation time) is the physical parameter of the graphene accounting for the intrinsic loss. Throughout this work, we assume *τ* = 0.2 ps which is in agreement with measured data from the chemical vapor deposited (CVD) graphene^44^. *T* is the room temperature and is fixed to 300 K in this paper. The sheet conductivity of graphene which can be derived using the well-known Kubo formula is described with interband and intraband contributions as^32^

3 $${\sigma }_{S}={\sigma }_{intra}(\omega ,{\mu }_{c},\Gamma ,T)+{\sigma }_{inter}(\omega ,{\mu }_{c},\Gamma ,T),$$

4 $${\sigma }_{intra}(\omega ,{\mu }_{c},\Gamma ,T)=-j\frac{{e}^{2}{k}_{B}T}{\pi {\hslash }^{2}(\omega -j2\Gamma )}(\frac{{\mu }_{c}}{{k}_{B}T}+2{\rm{In}}({e}^{-{\mu }_{c}/{k}_{B}T}+1)),$$

5 $${\sigma }_{{\rm{inter}}}(\omega ,{\mu }_{c},\Gamma ,T)\simeq \frac{-j{e}^{2}}{4\pi \hslash }{\rm{In}}(\frac{2|{\mu }_{c}|-(\omega -j2\Gamma )\hslash }{2|{\mu }_{c}|+(\omega -j2\Gamma )\hslash }),$$

where *e*, *ћ* and *k*_*B*_ are universal constants representing the electron charge, Planck’s and Boltzmann’s constant, respectively. If applying a DC bias voltage via the gated structure of the double layers of graphene grating, the Fermi level can be changed expediently, thus allows the control of the graphene conductivity. An approximate closed-form expression to relate *E*_F_ and *V*_*g*_ is given by^33^

6 $${E}_{F}\approx \hslash {\nu }_{f}\sqrt{\frac{\pi {\varepsilon }_{r}{\varepsilon }_{0}{V}_{g}}{e{t}_{s}}},$$

where *ε*_*r*_ and *ε*_0_ are the permittivity of iknsulating layer and vacuum respectively, *V*_*g*_ is the bias voltage, *e* and *ν*_*f*_ are the electron charge and the Fermi velocity (1.1 × 10^6^ m/s in graphene), respectively. Thus we can use the active metasurface to realize terahertz beam switching by electrically controlling the conductivity of graphene.
